# A Simple and High-Throughput ELISA-Based Neutralization Assay for the Determination of Anti-Flavivirus Neutralizing Antibodies

**DOI:** 10.3390/vaccines8020297

**Published:** 2020-06-10

**Authors:** Jean Claude Balingit, Minh Huong Phu Ly, Mami Matsuda, Ryosuke Suzuki, Futoshi Hasebe, Kouichi Morita, Meng Ling Moi

**Affiliations:** 1 Graduate School of Biomedical Sciences, Nagasaki University, Sakamoto 1-12-4, Nagasaki 852-8523, Japan; jcpbalingit@gmail.com (J.C.B.); minhhuong_ly@yahoo.com (M.H.P.L.); rainbow@nagasaki-u.ac.jp (F.H.); moritak@nagasaki-u.ac.jp (K.M.); 2 Department of Virology, Institute of Tropical Medicine, Nagasaki University, Sakamoto 1-12-4, Nagasaki 852-8523, Japan; 3 Program for Nurturing Global Leaders in Tropical and Emerging Communicable Diseases, Nagasaki University, Sakamoto 1-12-4, Nagasaki 852-8523, Japan; 4 Department of Virology II, National Institute of Infectious Diseases, 4-7-1 Gakuen, Musashi-murayama-shi, Tokyo 208-0011, Japan; mami@nih.go.jp (M.M.); ryosuke@nih.go.jp (R.S.); 5 Viet Nam Research Station, Center for Infectious Disease Research in Asia and Africa, Institute of Tropical Medicine, Nagasaki University, Sakamoto 1-12-4, Nagasaki 852-8523, Japan

**Keywords:** ELISA, microneutralization test, flavivirus, neutralizing antibodies, dengue, Zika

## Abstract

Mosquito-borne flavivirus infections, including dengue virus and Zika virus, are major public health threats globally. While the plaque reduction neutralization test (PRNT) is considered the gold standard for determining neutralizing antibody levels to flaviviruses, the assay is time-consuming and laborious. This study, therefore, aimed to develop an enzyme-linked immunosorbent assay (ELISA)-based microneutralization test (EMNT) for the detection of neutralizing antibodies to mosquito-borne flaviviruses. The inhibition of viral growth due to neutralizing antibodies was determined colorimetrically by using EMNT. Given the significance of Fcγ-receptors (FcγR) in antibody-mediated neutralization and antibody-dependent enhancement (ADE) of flavivirus infection, non-FcγR and FcγR-expressing cell lines were used in the EMNT to allow the detection of the sum of neutralizing and immune-enhancing antibody activity as the neutralizing titer. Using anti-flavivirus monoclonal antibodies and clinical samples, the utility of EMNT was evaluated by comparing the end-point titers of the EMNT and the PRNT. The correlation between EMNT and PRNT titers was strong, indicating that EMNT was robust and reproducible. The new EMNT assay combines the biological functional assessment of virus neutralization activity and the technical advantages of ELISA and, is simple, reliable, practical, and could be automated for high-throughput implementation in flavivirus surveillance studies and vaccine trials.

## 1. Introduction

Mosquito-borne viruses of the genus *Flavivirus* in the family *Flaviviridae*, including dengue virus (DENV) and Zika virus (ZIKV), are major public health threats in approximately a third of the world population that lives in transmission areas [1,2]. While the population at risk of these diseases is increasing, there are no effective antiflaviviral treatment approved for clinical use [3]. The development of an effective dengue vaccine has been hampered by limited understanding of the protection proxy against the disease [4]. Neutralizing antibody activity at protective levels plays a central role in flavivirus disease protection and disease development [5,6,7,8]. Hence, the determination of neutralizing antibodies that reflect biological activity is vital in the development of effective flavivirus vaccines. The gold standard for quantifying neutralizing antibody titers to flaviviruses is the plaque reduction neutralization test (PRNT) [9]. This method, however, is time-consuming, difficult to automate and low-throughput, representing a major bottleneck in conducting large-scale studies. PRNT is subject to analyst variability from manual plaque counting. In addition, as conventional PRNT uses cell lines that do not express FcγR receptors (FcγR) [10], the conventional method exclusively detects neutralization activity of antibodies but not the infection-enhancement activity (ADE activity) of antibodies. This limitation of the conventional PRNT has been highlighted in several studies, which hypothesized that the neutralizing titers as determined by FcγR-bearing cells better reflect the biological function of antibodies [11,12]. In this context, vaccine efficacy studies found that despite the induction of reasonable levels of neutralizing antibodies against four DENV serotypes, protection in some vaccinated participants was minimal [13,14,15].

A critical aspect regarding the evaluation of candidate dengue vaccines is the hypothetical risk of severe dengue due to non-protective cross-reactive ADE antibodies. ADE has been reported to occur in vitro [16,17,18,19,20] in vivo in animal models [20,21,22], and in dengue patients [23,24]. Hence, there is a need to address the discrepancies between seropositivity and vaccine efficacy outcomes [13,14,15]; emphasizing on the need for a reliable surrogate assay to determine an immune proxy that better reflects disease protection [25].

In this context, a simple, high-throughput neutralization test is also needed to adequately conduct large-scale surveillance studies and vaccine trials. The use of a microneutralization test (96-well plate format) offers a reliable alternative to the traditional PRNT—which is usually perfomed in 12-well or 24-well plate formats—as it is suitable for testing large numbers of samples. Several different microneutralization tests have been reported to be technically possible for mosquito-borne flaviviruses, such as the dengue virus [11,26,27,28,29,30,31] and Zika virus [28,30,31,32,33,34,35]. However, most of these tests require sophisticated equipment that is not always available in peripheral laboratories. These proposed methods may not be practical in countries with limited resources, and particularly when mosquito-borne flaviviral diseases are endemic in these areas. Hence, in the developing world, diagnostic and research laboratories need a simple, practical, economical, and reliable neutralization test which is useful in laboratories equipped for serology analyses.

In this study, the utility of an ELISA-based microneutralization test (EMNT) for the detection of neutralizing antibodies to mosquito-borne flaviviruses was developed and evaluated. In addition, cells that expresses the FcγR were used as assay cells in the EMNT to detect both neutralization activity and ADE activity as neutralizing antibody titer. The enzyme-linked immunosorbent assay (ELISA) format offers a practical approach as it is simple to perform and could be automated. Moreover, the ELISA read-out instrument is relatively inexpensive, and is an all-around device used not just for clinical diagnostics but for drug screening studies as well. The new test is useful in the detection of antibody neutralization activity to flaviviruses and could be applicable as a diagnostic technique that could contribute in seroepidemiological surveillance and evaluation of vaccine efficacy against flaviviruses.

## 2. Materials and Methods

### 2.1. Cells and Viruses

Baby hamster kidney cells (BHK-21, Japan Health Science Research Resource Bank) and African green monkey kidney Vero cells were maintained in Eagle’s minimum essential medium (EMEM) (Gibco, Gaithersburg, MD, USA) supplemented with heat-inactivated 10% fetal calf serum (FCS) without antibiotics. BHK-21 cells that expressed FcγRIIA, an activating FcγR, [36] were maintained in EMEM supplemented with heat-inactivated 10% FCS and 0.5 mg/mL neomycin (G418, PAA Laboratories GmbH, Pasching, Austria). Human embryonic kidney 293T cells were maintained in Dulbecco’s modified Eagle’s medium (Gibco) supplemented with non-essential amino acids, penicillin (100 U/mL), streptomycin (100 mg/mL), and heat-inactivated 10% FCS. All cell lines were cultured at 37 °C in a 5% CO_2_ incubator.

Dengue virus type-1 (DENV-1) 01-44-1HuNIID strain (GenBank accession no. AB111070), Dengue virus type-2 (DENV2) DHF0663 strain (GenBank accession no. AB189122), Dengue virus type-3 (DENV3) SLMC50 strain (GenBank accession no. GU377288), Dengue virus type-4 (DENV4) SLMC318 strain (GenBank accession no. KP893718), Zika virus (ZIKV) PRVABC59 strain (GenBank accession no. KX377337), Japanese encephalitis virus (JEV) OH0566 strain (GenBank accession no. AY508813) and yellow fever virus (YFV) 17D vaccine strain (GenBank accession no. NC_002031) were used. Viruses were propagated on BHK-21 cells at 37 °C in 5% CO_2_ for 5 days. Cell culture supernatant was collected, clarified by centrifugation, and stored in aliquots at −80 °C. DENV-4 SLMC318 was propagated on FcγRIIA-expressing BHK-21 in the presence of a mouse monoclonal IgG2a antibody (MAb HB-112 D1-4G2-4-15). Virus titers (plaque-forming units (PFU) per mL) were determined by plaque assay on BHK-21 cells.

### 2.2. Serum Samples and Monoclonal Antibodies

Serum samples were collected from confirmed dengue and Zika patients who resided in Ha Noi, northern Viet Nam, and in Central Vietnam between 2015–2018. A commercially available serum sample, obtained from a healthy donor (Human Serum Type AB, Lonza), was used as negative control serum. Serum samples were heat-inactivated at 56 °C for 30 min before use in the experiments. All sera used had been tested for DENV or ZIKV antibodies by IgG ELISA and IgM ELISA. Mouse monoclonal antibodies (200 µg/mL) that were flavivirus cross-reactive (IgG2a: MAb HB-112 D1-4G2-4-15 and 6B6C-1) and DENV-2 serotype-specific (IgG1: Mab HB-46 3H5-1) were also used for the neutralization assay.

### 2.3. Plaque Reduction Neutralization Test

Serial dilutions of heat-inactivated serum samples (serial 2-fold dilutions) were mixed with virus in a 1:1 ratio, and were incubated at 37 °C in 5% CO_2_ for 1 h. The virus-antibody mixture was inoculated in duplicate onto BHK-21 cells and FcγRIIA-expressing BHK-21 cells in 12-well plates. After adsorption for 1 h, 1.5 mL of overlay medium (1% methylcellulose with EMEM 2% FCS) was added to each well. Plates were incubated in 5% CO_2_ at 37 °C until plaques appeared. The cells were fixed with 4% paraformaldehyde in phosphate-buffered solution (Wako Pure Chemical Industries) for 1 hour at room temperature, and then stained with 0.25% crystal violet (Wako Pure Chemical Industries) overnight. Plaques were counted by naked eye, and the reciprocal serum dilution corresponding to the highest dilution with plaque counts less than 50% of the cut-off (≥50% inhibition) was considered the neutralizing titer.

### 2.4. ELISA-Based Microneutralization Test

An antigen-detection EMNT was performed in 96-well plates to measure virus neutralization. Conventional BHK-21 cells and FcγRIIA-expressing BHK-21 cells were seeded in 96-well plates at a density of 2 × 10^4^. Serial dilutions of heat-inactivated serum samples (serial 2-fold dilutions) were mixed with viruses at a 1:1 ratio and were incubated at 37 °C in 5% CO_2_ for 1 h. Each mixture was inoculated onto plates with cells and incubated at 37 °C in 5% CO_2_ for 1 h. Fresh medium was added and the plates were further incubated at 37 °C in 5% CO_2_ for 3 days. Each plate included both a virus control (no antibody) and a cell control (no virus, no antibody).

Three days after inoculation, culture supernatant was collected and an in-house antigen-detection ELISA was performed according to a previously described method [37]. Briefly, the polystyrene 96-well plates were coated with 100 µL/well (10 µg/mL) of anti-flavivirus immunoglobulin G (IgG) 12D11/7E8 [37] in ELISA coating buffer (0.05M carbonate-bicarbonate buffer, pH 9.6 containing 0.02% sodium azide) at 4 °C overnight. To avoid non-specific binding, wells were blocked with 100 µL of undiluted Block Ace UK-B 80 (Bio-Rad, Hercules, CA, USA) at room temperature for 1 h. Plates were washed five times with PBS containing 0.05% Tween 20 (PBS-T). Culture supernatant from the neutralization step was added into duplicate wells and incubated at 37 °C for 1 h. Plates were washed five times with PBS–T, and horseradish peroxidase (HRP)-conjugated 12D11/7E8 mouse monoclonal antibody [37] was added and incubated at 37 °C for 1 h. Plates were washed again five times with PBS–T, and HRP reaction was detected by adding *o*-phenylenediamine dihydrochloride (OPD) substrate (Sigma Chemicals) and 0.03% hydrogen peroxide in 0.05 M citrate-phosphate buffer, pH 5.0, for 30 min at room temperature away from light. The reaction was stopped with 1 N hydrochloric acid, and then the optical density (OD) was measured at 492 nm. The neutralizing titer was defined as the titer of the sample (antibody/serum) that reduced color development by 50% compared to the virus control wells.

### 2.5. Single-Round Infectious Particle Production and Neutralization Test

Single-round infectious particle (SRIP) production of the DENV-1 Yoyogi strain was performed as described previously [28]. Briefly, 293T cells were grown in a 90-mm dish and co-transfected with three plasmids: 2.5 µg of replicon plasmid, 1.25 µg of capsid-expression plasmid, and 1.25 µg of prME-expression plasmid, using Polyethylenimine Max (Cosmo-Bio, Tokyo, Japan). Culture medium was removed and replaced with fresh medium supplemented with 10 mM N-2-hydroxyethylpiperazine-N-ethanesulfonic acid (HEPES) buffer 2 days post-transfection. The medium was harvested after 3 days post-transfection and used as SRIPs. The infectious titer of generated SRIPs was determined by infection in Vero cells with subsequent luciferase assay.

SRIPs (50–100 infectious units/well) were used for the neutralization assay. Serial dilutions of monoclonal antibodies (serial 2-fold dilutions) were mixed with SRIPs in a 1:1 ratio and were incubated at 37 °C in 5% CO_2_ for 1 h. Vero cells were seeded in 96-well plates at a density of 1.2 × 10^4^. SRIP-antibody mixtures were inoculated onto plates with cells and incubated at 37 °C in 5% CO_2_ for 1 h. Fresh medium was added and plates were further incubated at 37 °C in 5% CO_2_ for 3 days. Each plate included both SRIP control (no antibody) and cell control (no SRIP, no antibody). The luciferase activity of cells was subsequently determined at 3 days post-infection using the Nano-Glo Luciferase Assay System (Promega, WI, USA). The neutralizing titer was determined as the antibody dilution that inhibited more than 50% of the SRIP inoculum without antibody (SRIP control).

### 2.6. Data Analysis

EMNT calculations were determined for each plate individually. Virus control wells should at least reach a median OD_492nm_ = 1.0–3.0, with the cell control at a low background median OD_492nm_ <0.2. Any sample well with an OD_492nm_ greater than twice the median OD_492nm_ of the cell control wells was considered positive; otherwise, it was considered negative. The OD_492nm_ cut-off of 50% virus neutralization for each plate was determined using the following equation [38]:

(1) $$x=\frac{median\;OD_{492\mathrm{nm}}\;of\;virus\;control\;wells+median\;OD_{492\mathrm{nm}}\;of\;cell\;control\;wells}{2}$$

Here, x is defined as 50% of the neutralization cut-off. The reciprocal antibody/serum dilution corresponding to the highest antibody/serum dilution with OD_492nm_ less than 50% of the cut-off (≥50% inhibition) was considered the neutralizing antibody titer for that sample.

Statistical analyses were performed using GraphPad Prism, version 8.2.1 (GraphPad, San Diego, CA, USA), with a 5% level of significance and two-tailed *p* values. Logarithmic transformation of the data were carried out to obtain an approximately normal distribution of the neutralizing titers. Data were tested for normal distribution using the Shapiro-Wilk test, and the correlation between EMNT and PRNT was determined using the Spearman correlation test.

### 2.7. Ethics Statement

This study was approved by the Institutional Review Board of the Institute of Tropical Medicine, Nagasaki University (EAN: 08061924-7). All participants provided their written informed consent to participate in this study.

## 3. Results

### 3.1. Development of the ELISA-Based Microneutralization Test

**Figure 1:**
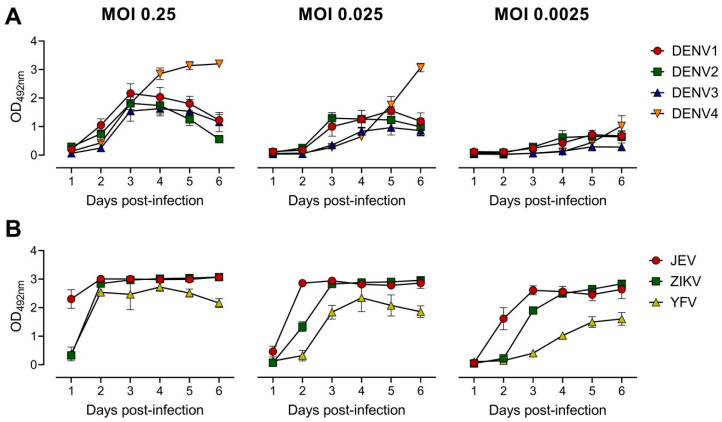
Quantitation of optical density (OD_492nm_) induced in BHK-21 cells post virus infection. BHK-21 cells were infected with virus at different MOIs as indicated. OD_492nm_ values were determined at 1 through 6 days post-infection. Growth curves of DENV 1–4 (**A**) and other flaviviruses: JEV, ZIKV and YFV (**B**) in BHK-21 cells were measured by antigen-detection ELISA [37]. Each data point represents the geometric mean value of duplicates ran independently thrice. Error bars depict standard deviation of six replicates.

To develop the EMNT, several parameters were tested in order to optimize the assay for sensitivity, reproducibility and efficiency. At first, the incubation time and challenge virus titer needed were optimized for the neutralization assay. Growth curves were established to determine the viral antigen production for representative mosquito-borne flaviviruses, namely: DENV1-4, ZIKV, JEV, and YFV. On a 96-well plate, BHK-21 cells were infected at a multiplicity of infection (MOI) of 0.25, followed by serial ten-fold dilutions up to 0.0025 for each virus. The growth curve between the first and sixth day after infection was determined to optimize the time point to recover cell culture supernatants for subsequent tests. At each time point, a total of 100 µL culture supernatant was collected and analyzed by antigen-detection ELISA [37]. The peak of viral antigen secretion generally occurred about three days after infection (Figure 1). In this study, a MOI of 0.25 in subsequent neutralization tests for DENV1-4, a MOI of 0.025 for ZIKV and YFV, and a MOI of 0.0025 for JEV was used. For each virus strain, the amount of optimal MOI that was used in the initial infection varied. The corresponding MOIs were approximately the highest dilution of virus that produced an OD of 1.0–3.0 in the antigen-detection ELISA after three days of incubation.

### 3.2. Determination of EMNT Titers Using Monoclonal Antibodies

After the optimization step, EMNT was performed by using mouse anti-E monoclonal antibodies with known neutralizing activities against flaviviruses. The OD in each well represents the amount of virus in the cell culture supernatant of BHK-21 or FcγRIIA-expressing BHK-21 cells, in the presence of serially diluted mouse monoclonal antibodies.

**Figure 2:**
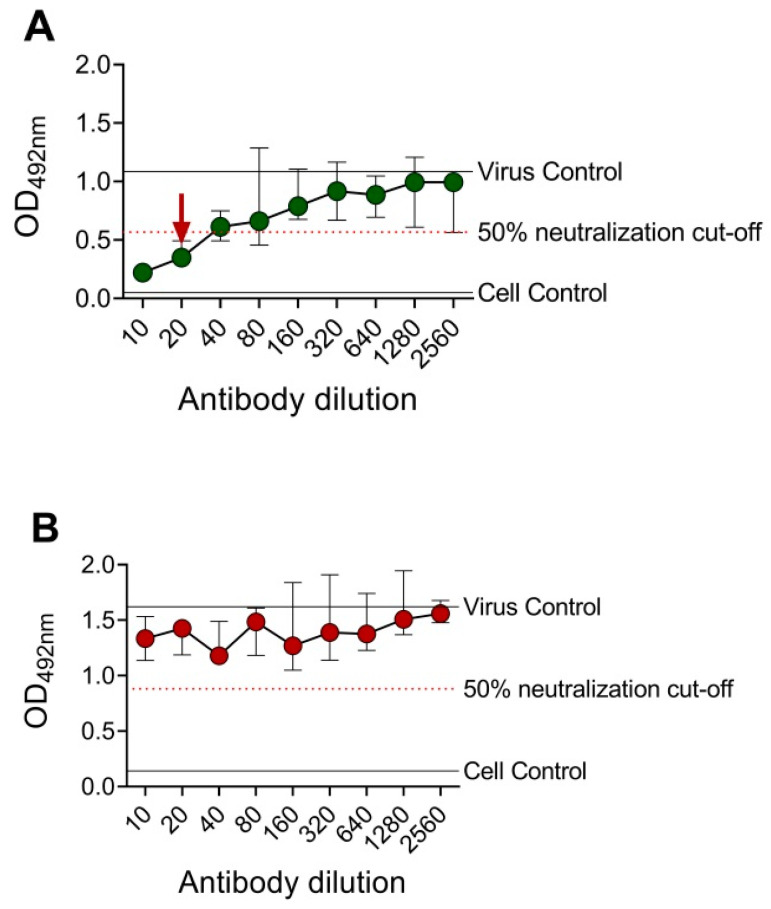
Sample EMNT results of an anti-E mouse monoclonal antibody tested against DENV-2. DENV-2 type-specific mouse monoclonal antibody, 3H5, was tested against DENV-2 DHF0663 using BHK-21 cells (**A**) and FcγRIIA-expressing BHK-21 cells (**B**). The neutralizing titer is the reciprocal of the highest antibody dilution that achieved ≥50% virus neutralization, as indicated by the arrow. No neutralization was observed when EMNT was performed using FcγRIIA-expressing BHK-21 cells. Each data point represents the median value of duplicates ran independently thrice. Error bars indicate 95% confidence interval of six replicates.

A DENV-2 serotype-specific mouse monoclonal antibody, 3H5, was tested against DENV-2 in BHK-21 cells and FcγRIIA-expressing BHK-21 cells (Figure 2). OD_492nm_ was plotted against the antibody dilutions, and the reciprocal of the highest antibody dilution that achieved ≥50% neutralization (EMNT_50_) was interpreted as the neutralizing titer. Consistent with the PRNT results, cross-reactive (4G2 and 6B6C-1) and DENV-2 serotype-specific (3H5) anti-E mouse monoclonal antibodies showed comparable neutralizing titers by using the EMNT (Table 1). Moreover, neutralizing titers to DENV serotypes as determined by BHK-21 cells were higher than those determined by FcγRIIA-expressing BHK-21 cells, which was consistent with a previous study [36].

### 3.3. Determination of EMNT Titers Using Clinical Samples

To evaluate the utility of EMNT, neutralization tests were performed with a panel of characterized clinical samples. This panel includes sera that were positive for either DENV IgG or ZIKV IgG. Neutralizing antibodies were detected against DENV1-4, JEV and ZIKV (Table 2 and Table 3). Most of the clinical samples demonstrated comparable levels of neutralizing titers or within 2- to 4-fold dilutions to that of the PRNT. Neutralizing titers to JEV or specific DENV serotype determined by BHK-21 cells were higher than those determined by FcγRIIA-expressing BHK-21 cells, and in some serum samples, neutralizing antibodies were not detected when FcγRIIA-expressing BHK-21 cells were used as the assay cells (Table 2). Some of the DENV IgG positive clinical samples exhibited cross-neutralization against JEV.

### 3.4. Comparison of EMNT to the PRNT

**Figure 3:**
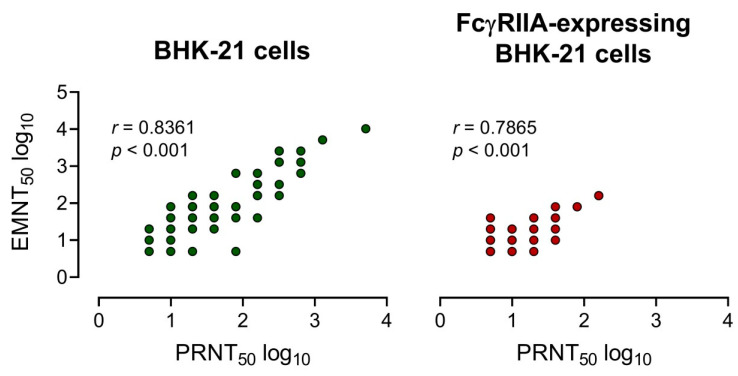
Correlation of neutralizing titers measured by EMNT vs PRNT. Correlation of the neutralizing titers of 25 serum samples (12 DENV IgG+ and 13 ZIKV IgG+ sera) and 3 anti-E mouse monoclonal antibodies determined by EMNT and PRNT using BHK-21 and FcγRIIA-expressing BHK-21 as assay cells. Neutralizing titers were expressed in log 10.

The neutralizing antibody titers of 25 serum samples [DENV IgG+: *n* = 12 and ZIKV IgG+: *n* = 13] and three mouse anti-E monoclonal antibodies were determined by using EMNT and PRNT. A strong correlation between EMNT titers and PRNT titers was obtained by using both BHK-21 cells or FcγRIIA-expressing BHK-21 cells, with a coefficient of determination (*r*) of 0.8361 and 0.7865, respectively (Figure 3). Additionally, there was a strong correlation between EMNT and PRNT to each DENV serotype, and to JEV and ZIKV (Table S1).

### 3.5. EMNT on a 384-Well Plate Format

**Figure 4:**
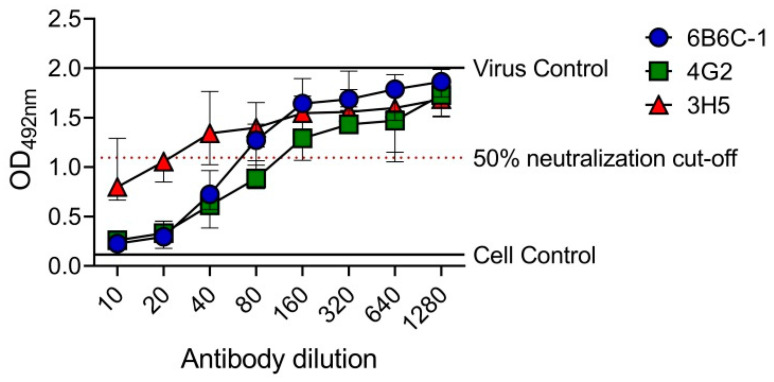
EMNT results of anti-E mouse monoclonal antibodies tested against DENV-2 performed on a 384-well plate. 6B6C-1, 4G2 and 3H5 were tested against DENV-2 DHF0663 on BHK-21 cells. The neutralization titer is the reciprocal of the highest antibody dilution that achieved ≥50% virus neutralization, as indicated by the arrow. Each data point represents the median value of four replicates. Error bars depict 95% confidence interval of four replicates.

To determine the utility of EMNT in a higher plate format, the EMNT was performed in a 384-well plate. In this plate format, the sample volume and reagent was reduced by two-folds (Table S2). Neutralizing antibody titers to DENV-2 by mouse monoclonal antibodies (6B6C-1, 4G2 and 3H5) was determined by BHK-21 cells. OD_492nm_ was plotted against the antibody dilutions, and the reciprocal of the highest antibody dilution that achieved ≥50% neutralization (EMNT_50_) was considered the neutralizing titer for the sample (Figure 4). The end-point titration values (EMNT_50_) using the 384-well plate format were consistent to those obtained using a 96-well plate format (Table 4). The result indicates that EMNT can be adapted to a 384-well plate format for neutralization tests.

### 3.6. EMNT Using Single-Round Infectious Particles (SRIPs)

**Figure 5:**
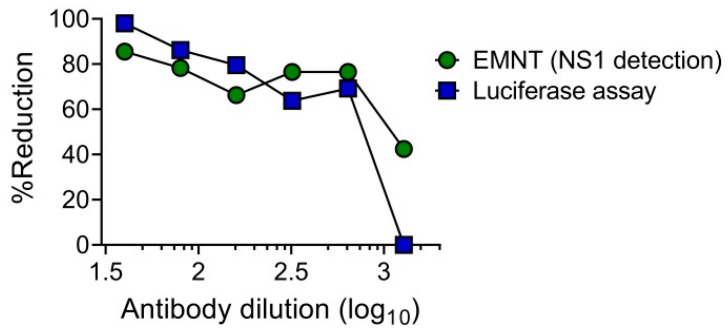
Comparison of EMNT and luciferase assay in detecting neutralizing titers to DENV1-SRIP using anti-flavivirus monoclonal antibody (6B6C-1). The monoclonal antibody was serially diluted two-fold and incubated with DENV1-SRIP at 37 °C for 1 h. The mixture was then titrated on Vero cells. A dose-dependent percentage reduction curve was obtained with the luciferase assay and EMNT.

The utility of single-round infectious particles (SRIPs) as an alternative to live viruses in neutralization tests has an advantage in terms of safety [39,40]. It has already been reported that SRIP [39,40] and reporter SRIP [28] can be used as an alternative in place of live virus in neutralization tests. These studies demonstrated that the dose-response curves obtained by using either SRIP or live virus were at similar levels. To determine the utility of SRIPs in the EMNT format, the neutralization test was performed by using DENV1-SRIP and Vero cells as assay cells, as previously described [28]. Neutralizing titer of an anti-flavivirus mouse monoclonal antibody, 6B6C-1 to DENV1 was determined by the EMNT format. Cell culture supernatants were harvested after 3 days incubation with SRIP-antibody mixture, and the levels of DENV NS1 antigen was determined by using EMNT based on a commercial ELISA kit (Platelia Dengue NS1 Ag kit (Bio-Rad)). The dose-response curve obtained using EMNT and the luciferase assay was consistent in both neutralization tests using 6B6C-1 (Figure 5). This result indicates that the DENV1-SRIP can be used in performing EMNT on Vero cells.

## 4. Discussion

Mosquito-borne flaviviruses are a major public health burden worldwide; hence, accurate diagnosis of flaviviral infections is vital for proper patient management. For diagnostic serology and evaluation of vaccine immunogenicity to flaviviruses, neutralization tests are extensively used to determine whether serum antibodies are able to inhibit virus infection either by blocking virus entry or preventing virus uncoating [41,42]. In this study, the novel EMNT detected not only neutralizing antibody activity to several mosquito-borne flaviviruses, but the test also detects the neutralizing titer in the presence of ADE activity by using FcγR-expressing assay cells. The EMNT yielded robust results that were consistent with the PRNT and was replicable in different cell lines. Furthermore, the EMNT method was applicable to both live viruses and SRIPs, highlighting the assay’s versatility and suitability in determining neutralizing titers to mosquito-borne flaviviruses.

The EMNT quantifies the amount of non-neutralized virus in the infected cell culture supernatant. In principle, the growth of non-neutralized virus is measured in a 96-well plate by using an antigen-detection ELISA system that uses a mouse anti-flavivirus envelope (E) monoclonal antibody [37]. The EMNT is based on the premise that the reduction in virus growth due to neutralization by antibodies can be measured optically by colorimetric changes. In this context, the approach to analyze viral antigen levels in the cell culture supernatant may be more effective than previously reported cell-based ELISA microneutralization assays [26,35] as cells can be flushed from the plate during multiple plate washings, resulting in lower OD values and hence lower sensitivity.

Like the PRNT, the EMNT end-point titers could be determined for a clinical sample at any selected percent reduction of virus activity. A potential advantage of this new test is eliminating the bottleneck of visualization of plaques that must be large enough to be visible to the naked eye. The PRNT measures the presence of visible plaques, whereas the EMNT quantifies the amount of viral antigen secreted to the cell culture supernatant. The integration of colorimetric detection and automated counting of OD values allows rapid detection; from 5–7 days incubation with the PRNT to 3 days with the EMNT. Because the readout does not require laborious manual plaque counting, the EMNT is highly relevant in high-throughput screening of large numbers of sera for neutralizing activity. Notably, as not all clinical isolates produce clear plaques, the PRNT method is applicable only to virus strains that are capable of forming plaques. The EMNT circumvents the need for visualizing plaques as the test detects the viral antigen secreted to the cell culture supernatant.

To evaluate the performance of EMNT on representative mosquito-borne flaviviruses we used anti-flavivirus mouse monoclonal antibodies and characterized clinical samples. Using an in-house ELISA method [37], the results suggest that the EMNT was technically easy to perform as compared to the PRNT and the results were comparable to that of the PRNT. The higher titers and titer increases detected by the EMNT in comparison to PRNT contrasts with the findings of a previous study that also used an ELISA-based format [26]. However, it should be noted that the EMNT of ZIKV and YFV using mouse monoclonal antibodies (Table 1) showed substantially higher neutralizing titers (≥4 fold) than the PRNT which did not demonstrate neutralization activity, indicating that virus secretion mechanism could differ from that of plaque formation. However, the ZIKV-EMNT on BHK-21 cells using sera from individuals with confirmed ZIKV infection (Table 3) demonstrated a strong correlation between the ZIKV-EMNT and the ZIKV-PRNT (Supplementary Table S1). Serum samples from DENV IgG+ patients neutralized JEV at comparable levels to that of DENV. These results confirm the presence of cross-reactive neutralizing activity between DENV and JEV in these patients [43,44]. In the sampling site (Vietnam) wild-type JEV continues to co-circulate with DENV, and JEV vaccination coverage in this region is high [45,46]. For this reason, significant cross-reactivity was observed between DENV and JEV from DENV patients.

In the context of assessing vaccine immunogenicity, a major advantage of the EMNT may be the better prediction of in vivo protection from flavivirus infections as this test can be adapted to use FcγR-bearing cells as assay cells. FcγR-bearing monocytes have been demonstrated to be major target cells for DENV infection and replication in vivo [47,48]. Given the significance of FcγR in mediating neutralization and ADE especially in DENV infection, the use of FcγR-bearing cells as assay cells in EMNT allows the determination of the biological properties of anti-flavivirus antibodies with both neutralizing and ADE activity and thus, offers a correlate that better reflect protection against mosquito-borne flavivirus infections. The results on the DENV IgG+ clinical samples demonstrated that the DENV-EMNT performed on conventional BHK-21 cells (non-FcγR cells) showed higher neutralizing titers as compared to the FcγRIIA-expressing BHK-21 cells. These results are consistent with previous studies in which DENV neutralizing titers were higher in FcγR-negative cells [11,49,50,51].

To examine the correlation between the EMNT and the PRNT, the neutralizing titers of three mouse monoclonal antibodies and 25 characterized clinical samples using both tests was used. For BHK-21 cells and FcγRIIA-expressing BHK-21 cells, the EMNT end-point titers (EMNT_50_) correlated with the PRNT end-point titers (PRNT_50_), with a coefficient of determination (*r*) of 0.8361 and 0.7865, respectively, suggesting a strong correlation between the EMNT and the PRNT. The EMNT was concordant with the PRNT for each virus on a given cell line and neutralizing titer (Table S1). Collectively, these results demonstrate that the EMNT maintains the reliability of the PRNT.

The results also suggest that the 384-well format EMNT is suitable for use in detecting neutralizing titers, and that the smaller volume of the 384-well plate did not affect the ability of the EMNT to detect neutralizing titers. Further reduction in sample and reagent volumes may be possible in future experiments with optimization. The capability of the 384-EMNT to use lesser amounts of reagents and samples is especially important if higher-throughput neutralization tests will contribute to a faster turnaround time in surveillance and vaccine trials. The development of large-scale neutralization tests for flaviviruses would enable larger numbers of serum samples to be tested in follow-up studies on currently available vaccines and would also allow more rapid routine surveillance on naturally occurring protective immunity against flaviviral infections. In addition, a robotic platform for high-throughput determination of neutralizing titers allows the rapid evaluation of large numbers of samples, which is worth considering especially in conducting vaccine trials. The use of a robotics system in the EMNT would provide an opportunity to rapidly evaluate vast numbers of samples, hence increasing the efficiency of screening for neutralizing antibodies.

In this study, the utility of SRIPs in place of live viruses was also evaluated using the EMNT format. In SRIPs, the absence of the structural gene in the packaged genome allows SRIP-infected cells to produce non-infectious progeny viruses [28]. To determine the levels of the SRIP in the cell culture supernatant, the levels of non-structural protein 1 (NS1) in the cell culture supernatant of SRIP-infected cells was determined. Neutralizing titers were at comparable levels between the SRIP-luciferase assay and the EMNT. The results suggest that the EMNT format was useful in the detection of neutralizing antibodies by using either live virus strains or SRIPs. Of note, the EMNT, like the PRNT, is a cell-based assay associated with high variability where intra-assay variability is high especially when the same sample is tested on different days. However, unlike the PRNT, the ELISA system was used to detect neutralizing titers instead of manual plaque count, hence, the ELISA OD results is not subjected to plaque formation and variability due to clinical strains, which can lead to difficulties in plaque visualization and results interpretation.

## 5. Conclusions

A novel test based on the EMNT method was developed to determine the sum of neutralizing antibody titer in the presence of ADE activity to representative mosquito-borne flaviviruses. There was a strong correlation between the EMNT and the PRNT, indicating that the newly developed test is robust, replicable and could be used as a high-throughput alternative to the PRNT. The new test has potential value as a basic research and diagnostic tool that could be used to fast-track the throughput of neutralization assays for seroepidemiological investigations and vaccine studies. As EMNT is ELISA-based, the test could also be translated as an in vitro immunoassay kit that could aid in standardizing the performance of neutralization tests for multiple flaviviruses. More importantly, due to the simplicity of the EMNT and as both live viruses and SRIPs could be ultilized, the new test has the potential to play a critical role in improving capacity for diagnosis and routine surveillance of flaviviral infections, particularly in the developing world.

## Figures and Tables

**Table 1:** Neutralizing Titers of Anti-E Monoclonal Antibodies (EMNT_50_ vs. PRNT_50_) to DENV, JEV, ZIKV and YFV Using BHK-21 and FcγRIIA-Expressing BHK-21 as Assay Cells.

Virus	Flavivirus Cross-Reactive Monoclonal Antibody								DENV-2 Type-Specific Monoclonal Antibody			
	mAb 6B6C-1				mAb 4G2				mAb 3H5			
	BHK-21		FcγR-BHK-21		BHK-21		FcγR-BHK-21		BHK-21		FcγR-BHK-21	
	EMNT ^a^	PRNT ^b^	EMNT	PRNT	EMNT	PRNT	EMNT	PRNT	EMNT	PRNT	EMNT	PRNT
DENV-1	40	40	10	<10 ^c^	40	40	10	<10	<10	<10	<10	<10
DENV-2	80	40	20	10	80	80	10	10	20	40	<10	<10
DENV-3	20	10	<10	<10	10	10	<10	<10	<10	<10	<10	<10
DENV-4	40	20	<10	<10	40	80	<10	<10	<10	<10	<10	<10
JEV	20	<10	20	<10	20	<10	10	<10	<10	<10	<10	<10
ZIKV	80	<10	80	<10	80	<10	40	<10	<10	<10	<10	<10
YFV	320	<10	40	<10	160	<10	80	<10	<10	<10	<10	<10

^a^ EMNT_50_ end points were determined by using the reciprocal of the final antibody dilution that reduced color development (OD_492nm_) by 50% compared to the virus control wells (no antibodies); ^b^ PRNT_50_ end points were determined by using the reciprocal of the final antibody dilution showing ≥50% reduction in plaque counts in test wells compared to the number of plaques from the virus control wells (no antibodies); ^c^ Titers less than 10 (<10) indicates neutralization titers below the detection limit of the assay. Samples were serially diluted two-fold from 1:10 to 1:2560.

**Table 2:** Neutralizing Titers of 12 DENV IgG+ Clinical Samples Against DENV and JEV Using EMNT and PRNT by Using BHK-21 Cells and FcγRIIA-Expressing BHK-21 Cells as Assay Cells.

Sample Code	DENV-1				DENV-2				DENV-3				DENV-4				JEV			
	BHK-21		FcγR-BHK-21		BHK-21		FcγR-BHK-21		BHK-21		FcγR-BHK-21		BHK-21		FcγR-BHK-21		BHK-21		FcγR-BHK-21	
	EMNT ^a^	PRNT ^b^	EMNT	PRNT	EMNT	PRNT	EMNT	PRNT	EMNT	PRNT	EMNT	PRNT	EMNT	PRNT	EMNT	PRNT	EMNT	PRNT	EMNT	PRNT
HN.15.001/1	20	<10 ^c^	20	<10	<10	<10	<10	<10	<10	10	<10	<10	80	20	<10	<10	320	320	160	160
HN.15.018/1	<10	<10	10	10	20	40	<10	<10	<10	<10	<10	<10	160	40	<10	<10	20	40	<10	10
HN.15.022/1	40	20	40	<10	80	80	10	40	20	40	10	<10	80	20	<10	<10	80	20	<10	<10
HN.15.056/1	20	40	40	20	10	<10	<10	<10	<10	<10	<10	<10	20	<10	<10	<10	160	160	80	80
HN.15.068/1	<10	20	10	10	80	80	<10	10	<10	<10	<10	<10	80	20	<10	<10	40	40	<10	<10
HN.15.071/1	<10	10	<10	<10	80	80	<10	10	<10	<10	<10	<10	40	10	<10	<10	<10	<10	<10	<10
HN.15.082/1	320	160	160	160	20	40	10	20	160	160	40	40	160	20	10	<10	160	160	80	40
HN.15.084/1	640	160	160	160	<10	<10	<10	<10	160	160	<10	<10	80	10	<10	<10	<10	<10	<10	<10
HN.15.086/1	160	160	160	160	40	160	20	40	80	80	20	10	320	160	20	20	40	20	<10	<10
HN.15.097/1	640	320	160	160	40	40	40	40	160	160	80	80	320	160	20	10	40	20	10	10
HN.15.011/1	640	640	160	160	80	40	40	20	*NT*	*NT*	*NT*	*NT*	*NT*	*NT*	*NT*	*NT*	640	160	40	40
HN.15.026/1	20	40	<10	<10	160	160	<10	20	*NT*	*NT*	*NT*	*NT*	*NT*	*NT*	*NT*	*NT*	<10	<10	<10	<10

^a^ EMNT_50_ end points were determined by using the reciprocal of the final antibody dilution that reduced color development (OD_492nm_) by 50% compared to the virus control wells (no antibodies); ^b^ PRNT_50_ end points were determined by using the reciprocal of the final antibody dilution showing ≥50% reduction in plaque counts in test wells compared to the number of plaques from the virus control wells (no antibodies); ^c^ Titers less than 10 (<10) indicates neutralization titers below the detection limit of the assay. Samples were serially diluted two-fold from 1:10 to 1:1280.

**Table 3:** Neutralizing Titers of 13 ZIKV IgG Positive Clinical Samples to ZIKV by Using BHK-21 Cells.

	Neutralizing Titers to ZIKV	
Sample Code	EMNT ^a^	PRNT ^b^
Z67 SRII	2560	640
Z68 SRII	2560	320
Z77	1280	320
Z78	10240	5120
Z79	1280	640
Z84	2560	320
Z78H2	5120	1280
Z79H2	1280	320
Z120	<20 ^c^	<20
Z123	640	80
Z125	40	20
Z126	20	20
Z129	40	40

^a^ EMNT_50_ end points were determined by using the reciprocal of the final serum dilution that reduced color development (OD_492nm_) by 50% compared to the virus control wells (no sera); ^b^ PRNT_50_ end points were determined by using the reciprocal of the final serum dilution showing a 50% or greater reduction in plaque counts in wells compared to the number of plaque from the virus control wells (no sera); ^c^ Titers less than 20 (<20) indicates neutralization titers below the detection limit of the assay. Samples were serially diluted two-fold from 1:20 to 1:10,240.

**Table 4:** Neutralizing Titers of Anti-E Monoclonal Antibodies (EMNT_50_) to DENV-2 Using BHK-21 Cells on Both 96-Well and 384-Well Plates.

Monoclonal Antibody	384-well	96-well
6B6C-1	40	80
4G2	80	80
3H5	20	20

## Supplementary Materials

The following are available online at https://www.mdpi.com/2076-393X/8/2/297/s1, Table S1: Correlation coefficients between EMNT and PRNT for each virus on BHK-21 cells and FcγRIIA-expressing BHK-21 cells. Table S2. EMNT conditions on both 96-well and 384-well plates.
