# Supplementary material for: A Simple and High-Throughput ELISA-Based Neutralization Assay for the Determination of Anti-Flavivirus Neutralizing Antibodies
Source: Vaccines (Basel). 2020 Jun 10;8(2):297. doi: 10.3390/vaccines8020297 (PMC7350015; doi:10.3390/vaccines8020297)
Supplement: Supplementary file 1 [file vaccines-08-00297-s001.pdf]

Table S1. Correlation coefficients between EMNT and PRNT for each virus on BHK-21 cells and FcγRIIA-expressing BHK-21 cells.

**Virus**	**Cell line**	**Spearman's Correlation Coefficient (*r*)**	**95% CI**	***p* Values (Two-Tailed)**
ZIKV	BHK-21	0.8573	0.6189 – 0.9510	$p < 0.001$
JEV	BHK-21	0.8592	0.6989 – 0.9661	$p < 0.001$
JEV	FcγR-BHK-21	0.7890	0.4512 – 0.9290	$p < 0.001$
DENV-1	BHK-21	0.8603	0.6120 – 0.9542	$p < 0.001$
DENV-1	FcγR-BHK-21	0.8117	0.5001 – 0.9372	$p < 0.001$
DENV-2	BHK-21	0.7891	0.4515 – 0.9291	$p < 0.001$
DENV-2	FcγR-BHK-21	0.7298	0.3327 – 0.9071	$p = 0.003$
DENV-3	BHK-21	0.9517	0.8370 – 0.9863	$p < 0.001$
DENV-3	FcγR-BHK-21	0.9031	0.6914 – 0.9720	$p < 0.001$
DENV-4	BHK-21	0.7446	0.3119 – 0.9215	$p = 0.004$
DENV-4	FcγR-BHK-21	0.8469	0.5419 – 0.9548	$p < 0.001$

Table S2. EMNT conditions on both 96-well and 384-well plates.

Assay Conditions			384-Well	96-Well
I. Neutralization	Cell seeding density		5×10^3 cells/well	2×10^4 cells/well
	Virus-antibody mixture for infection		25 μL/well	50 μL/well
	Fresh medium added after infection		62.5 μL	125 μL
II. Antigen-detection ELISA	Coating antibody	12D11/7E8 (10 μg/mL)	50 μL	100 μL
	Sample from neutralization step (I)	Virus-antibody mixture	50 μL	100 μL
	Detecting antibody	HRP-conjugated 12D11/7E8 (1:1000)	50 μL	100 μL
	Color development	OPD in substrate buffer with 0.03% H_2O_2	50 μL	100 μL
	Stop reagent	HCl	25 μL (2N)	100 μL (1N)
